# Baseline serum syndecan-4 predicts prognosis after the onset of acute exacerbation of idiopathic interstitial pneumonia

**DOI:** 10.1371/journal.pone.0176789

**Published:** 2017-05-03

**Authors:** Yuki Sato, Yoshinori Tanino, Xintao Wang, Takefumi Nikaido, Suguru Sato, Kenichi Misa, Ryuichi Togawa, Charles W. Frevert, Mitsuru Munakata

**Affiliations:** 1 Department of Pulmonary Medicine, Fukushima Medical University School of Medicine, Fukushima, Fukushima, Japan; 2 Division of Pulmonary/Critical Care Medicine, Department of Comparative Medicine, University of Washington, Seattle, Washington, United States of America; 3 The Comparative Pathology Program in the Department of Comparative Medicine, University of Washington, Seattle, Washington, United States of America; 4 The Center of Lung Biology, University of Washington, Seattle, Washington, United States of America; Keio University, JAPAN

## Abstract

**Background:**

Patients with idiopathic interstitial pneumonia can experience acute respiratory worsening, also known as acute exacerbation, with a large deterioration on prognosis. The precise mechanism remains unclear; however, syndecan-4 may be involved. Syndecan-4, a transmembrane heparan sulfate proteoglycan expressed in a variety of cells (e.g., epithelial cells, macrophages, fibroblasts, etc.), performs various biological roles by binding to several proteins through its heparan sulfate glycosaminoglycan side chains. The goal of this study was to clarify the role of syndecan-4 in acute exacerbation of idiopathic interstitial pneumonia.

**Methods:**

Patients with idiopathic interstitial pneumonia who had been sequentially admitted to our hospital due to acute exacerbation were retrospectively analyzed. First, serum syndecan-4 levels in the acute exacerbation and clinically stable phases were compared. Second, the relationship between serum syndecan-4 levels and clinical parameters was analyzed. Third, the relationship between serum syndecan-4 levels and prognosis was evaluated.

**Results:**

Serum syndecan-4 levels were significantly lower in patients with acute exacerbation of idiopathic interstitial pneumonia than in patients in the clinically stable phase. Serum syndecan-4 levels also showed a significant positive correlation with white blood cell count and a weak positive tendency with KL-6 and baseline %VC. Prognosis was significantly worse in patients with idiopathic interstitial pneumonia with high baseline serum syndecan-4 levels than with low baseline levels. Multiple logistic analysis indicated baseline serum syndecan-4 level as the only prognostic predictor following acute exacerbation.

**Conclusions:**

Baseline serum syndecan-4 is a possible prognostic biomarker after the onset of acute exacerbation of idiopathic interstitial pneumonia.

## Introduction

Idiopathic pulmonary fibrosis (IPF) is a chronic, progressive, and intractable fibrosing lung disease that can progress to rapid respiratory failure during the disease course. IPF of unknown cause and without symptoms of left heart failure, pulmonary embolism, or lung infection is referred to as acute exacerbation (AE) of IPF [1–8]. The reported frequency of AE is 1, 2, or 3 years, at rates of 8.6%, 12.6%, and 23.9%, respectively [9]. The post-onset mortality rate is approximately 50%, so IPF has an extremely poor prognosis [10, 11]. AE accounts for 40% of all IPF-related deaths [12], making it a critical event during the clinical course of IPF. However, the pathogenesis of AE is poorly understood, and no standard treatment is yet available.

One potential factor that may contribute to the pathogenesis of AE of interstitial pneumonia is the proteoglycan syndecan-4. Proteoglycans are glycoproteins composed of a core protein associated with one or more side chain glycosaminoglycans, which are highly negatively charged sulfated polysaccharides. The syndecans are a family of proteoglycans associated with three to five heparan sulfate or chondroitin sulfate side chains. They are transmembrane proteins and are expressed on the surfaces of many cells. The syndecan family in humans has four members—syndecan-1 to syndecan-4—that have reported involvement in lung development, wound healing, and inflammatory reactions [13]. Syndecans exist in cell surface forms, as well as in soluble forms that are shed from the cell membrane by matrix metalloproteinases (MMPs) [14–16]. The heparan sulfate side chains are thought to bind to various cytokines, chemokines, and growth factors, thereby regulating biological activity [17].

Our previous comparison of syndecan-4-deficient mice and wild-type mice indicated that neutrophil migration and injury are increased in the lungs of syndecan-4-deficient mice after intratracheal instillation of lipopolysaccharide (LPS) [18]. We also found higher serum syndecan-4 levels in hospitalized patients with bacterial pneumonia than in healthy subjects, and the syndecan-4 levels tended to increase over the course of pneumonia in those patients with a favorable prognosis [19]. Another effect of syndecan-4 may involve its interaction with the C-X-C motif chemokine ligand 10 (CXCL10), which shows an anti-fibrotic effect upon binding to syndecan-4 in a bleomycin-induced mouse model of pulmonary fibrosis [20]. These results suggest an important role for syndecan-4 in limiting lung inflammation and fibrosis. The aim of the present study was to clarify the role of syndecan-4 in AE of idiopathic interstitial pneumonia (IIP).

## Materials and methods

### Subjects

The ethics committee of Fukushima Medical University approved this work (approval number: 1105), and all clinical investigation were conducted according to the principles of the Declaration of Helsinki. Informed consent was not obtained because the data were analyzed anonymously. Patients who were sequentially hospitalized for AE of IIP (AE-IIP) at our department between 2007 and 2014, patients with clinically stable IIP (SD-IIP) without subjective symptoms of dyspnea or rapid deterioration on image findings for at least 3 months, and healthy volunteers (HV) were included in this retrospective analysis. The diagnostic criteria for AE were set according to a definition of AE in a previous report [21]. The criteria for inclusion were as follows: progression of dyspnea within the past month; new bilateral infiltrative shadows or ground glass opacities on high-resolution computed tomography (HRCT); and a decrease in partial pressure of arterial oxygen (PaO_2_) of at least 10 torr or a PaO_2_/fraction of inspiratory oxygen (FiO_2_) (P/F) ratio of < 300 mmHg. Patients with pneumonia, heart failure, pulmonary embolism, and pneumothorax were excluded. Patients whose progression of pulmonary fibrosis was clearly associated with another disease (collagen disease, drug-induced lung injury, pneumoconiosis, hypersensitivity pneumonitis, sarcoidosis, pulmonary Langerhans cell histiocytosis, lymphangioleiomyomatosis, etc.) were also excluded. IPF was diagnosed using the definition in the 2011 ATS/ERS/JRS/ALAT joint statement [22], and all patients with IIP met the 2013 ATS/ERS Update of the International Multidisciplinary Classification of the IIP [23]. Diagnosis of systemic inflammatory response syndrome (SIRS) [24] and APACHE II classifications [25] were made according to past diagnostic criteria at the time of admission. All patients underwent steroid pulse therapy as treatment.

### Measurement of serum syndecan-4

We first compared serum syndecan-4 levels in patients with AE-IIP and SD-IIP, as well as in HV. Syndecan-4 levels were measured as described previously [19] with a commercially available ELISA kit (IBL, Takasaki, Japan) according to the manufacturer’s protocol.

### The relationship between serum syndecan-4 and clinical parameters

We also examined the relationship between serum syndecan-4 levels and clinical characteristics, including blood laboratory findings and HRCT findings upon admission.

### The relationship between serum syndecan-4 and prognosis

We compared the clinical data of a survival group and a non-survival group, as defined at 60 days after admission for AE. We also used univariate and multivariate analysis to evaluate prognostic factors, and we used Kaplan-Meier analysis to examine the relationship between syndecan-4 level and prognosis.

### Statistical analysis

Data were expressed as means ± the standard error of the mean (SEM). Two unpaired groups were compared with the Mann-Whitney U test or Fisher’s exact test, while multiple groups were compared with ANOVA with the Tukey HSD. Serum syndecan-4 levels in each IIP patient during AE and during the stable phases (baseline) were analyzed with Wilcoxon’s signed-rank test. Correlations between serum syndecan-4 levels and clinical parameters were analyzed using Spearman’s correlation coefficient and survival predictors 60 days after admission were subjected to logistic regression analysis in the univariate and multivariate analyses. Prognoses according to syndecan-4 levels were compared with a log-rank test. The analysis was performed using IBM SPSS Statistics 17.0 software (IBM Japan; Tokyo, Japan), and a p value of < 0.05 was considered statistically significant.

## Results

### Clinical characteristics

Fifty-six AE-IIP patients were hospitalized during the study period; 21 were patients with IPF and 35 were patients with IIP other than IPF (non-IPF). The patients had a mean age of 70 ± 1 years, and included 47 men and nine women. The AE-IIP patients had a reduced P/F ratio of 206 ± 16 mmHg, as well as elevated KL-6, surfactant protein (SP)-A, and SP-D levels of 1524 ± 127 U/mL (reference < 500 U/mL), 119 ± 7 ng/mL (reference < 43.8 ng/mL), and 344 ± 53 ng/mL (reference < 110 ng/mL), respectively (Table 1). A comparison of the AE-IIP and SD-IIP patients revealed a significantly higher white blood cell (WBC) count, significantly higher lactate dehydrogenase (LDH), SP-A, and C-reactive protein levels, and a higher erythrocyte sedimentation rate in the AE-IIP patients. The levels of SP-D also tended to be higher in the AE-IIP patients.

**Table 1:** Clinical characteristics of healthy volunteers, patients with AE-IIP, and patients with SD-IIP.

	Healthy Volunteers	Patients With SD-IIP	Patients With AE-IIP	P Value
Subjects (n)	45	62	56	
Age (years)	43±2*^†^	68±1	70±1	<0.01
Gender (M/F)	29/16	47/15	47/9	0.078
IPF/non IPF (n)	NA	33/29	21/35	0.063
Duration from diagnosis to hospitalization for AE (months)	NA	NA	24±4	
Duration of symptoms before admission (days)	NA	NA	9±1	
Survivor /Non-survivor at 60 days after admission (n)	NA	NA	25/31	
Glucocorticoid pulse therapy (n)	NA	NA	56	
Cyclophosphamide pulse therapy (n)	NA	NA	7	
Sivelestat (n)	NA	NA	23	
WBC (/μl)	NA	7907±315	10439±618	0.001
LDH (IU/L)	NA	260±8	450±56	<0.001
KL-6 (U/ml)	NA	1463±144	1524±127	0.485
SP-A (ng/ml)	NA	98±8	119±7	0.012
SP-D (ng/ml)	NA	250±24	344±53	0.084
CRP (mg/dl)	NA	1.36±0.48	7.97±0.88	<0.001
ESR (mm/h)	NA	28±3	39±4	0.012
PCT (ng/ml)	NA	NA	0.65±0.27	
BNP (pg/ml)	NA	NA	90.1±21.2	
FBG (mg/dl)	NA	NA	453±16	
FDP (μg/ml)	NA	NA	13.7±3.5	
D-dimer (μg/ml)	NA	NA	6.6±1.6	
P/F on admission (mmHg)	NA	NA	206±16	
SIRS (+/-)	NA	NA	34/22	
SIRS score	NA	NA	1.9±0.1	
APACHE II score	NA	NA	13.7±0.7	
VC (L)	NA	2.55±0.18	NA	
%VC (%)	NA	79.8±4.8	NA	
Serum syndecan-4 (ng/ml)	16.05±0.77	25.22±3.72*^ǂ^	10.65±0.73	<0.001

SD: stable disease, AE: acute exacerbation, IPF: idiopathic pulmonary fibrosis, WBC: white blood cell count, LDH: lactate dehydrogenase, KL-6: Krebs von den Lungen-6, SP-A: surfactant protein-A, SP-D: surfactant protein-D, CRP: c-reactive protein, ESR: erythrocyte sedimentation rate, PCT: procalcitonin, BNP: brain natriuretic peptide, FBG: fibrinogen, FDP: fibrin and fibrinogen degradation product, P/F: arterial partial pressure of carbon dioxide/Fraction of inspiratory oxygen, SIRS: systemic inflammatory response syndrome, VC: vital capacity. APACHE II score: acute physiology and chronic health evaluation II score.

* Healthy Volunteers vs Patients with SD-IIP.

†Healthy Volunteers vs Patients with AE-IIP.

ǂ Patients with SD-IIP vs Patients with AE-IIP. Mean ± SEM.

### Serum syndecan-4 levels in AE-IIP patients

**Fig 1:**
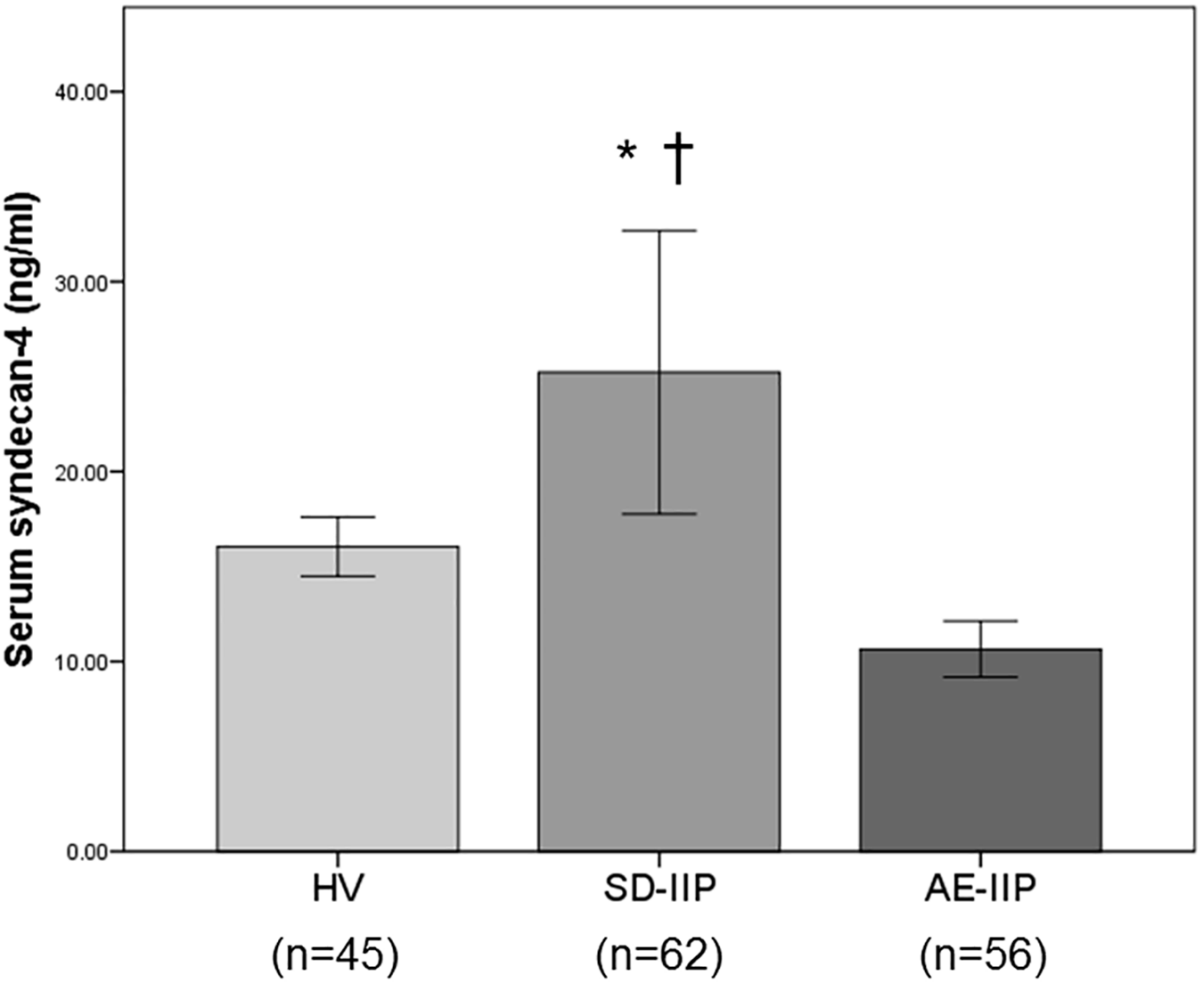
Comparison of serum syndecan-4 levels in healthy volunteers and in patients with SD-IIP and AE-IIP. Serum syndecan-4 levels were significantly higher in the SD-IIP group than in the HV and AE-IIP groups. Serum syndecan-4 levels did not differ between the HV and AE-IIP groups. HV: healthy volunteer group, SD-IIP: patient group with stable idiopathic interstitial pneumonia, AE-IIP: patient group with acute exacerbation of idiopathic interstitial pneumonia. ANOVA with Tukey HSD was used for statistical analysis. *: p < 0.05 vs HV. †: p < 0.001 vs AE-IIP. Data are expressed as means ± the standard error of the mean (SEM).

**Fig 2:**
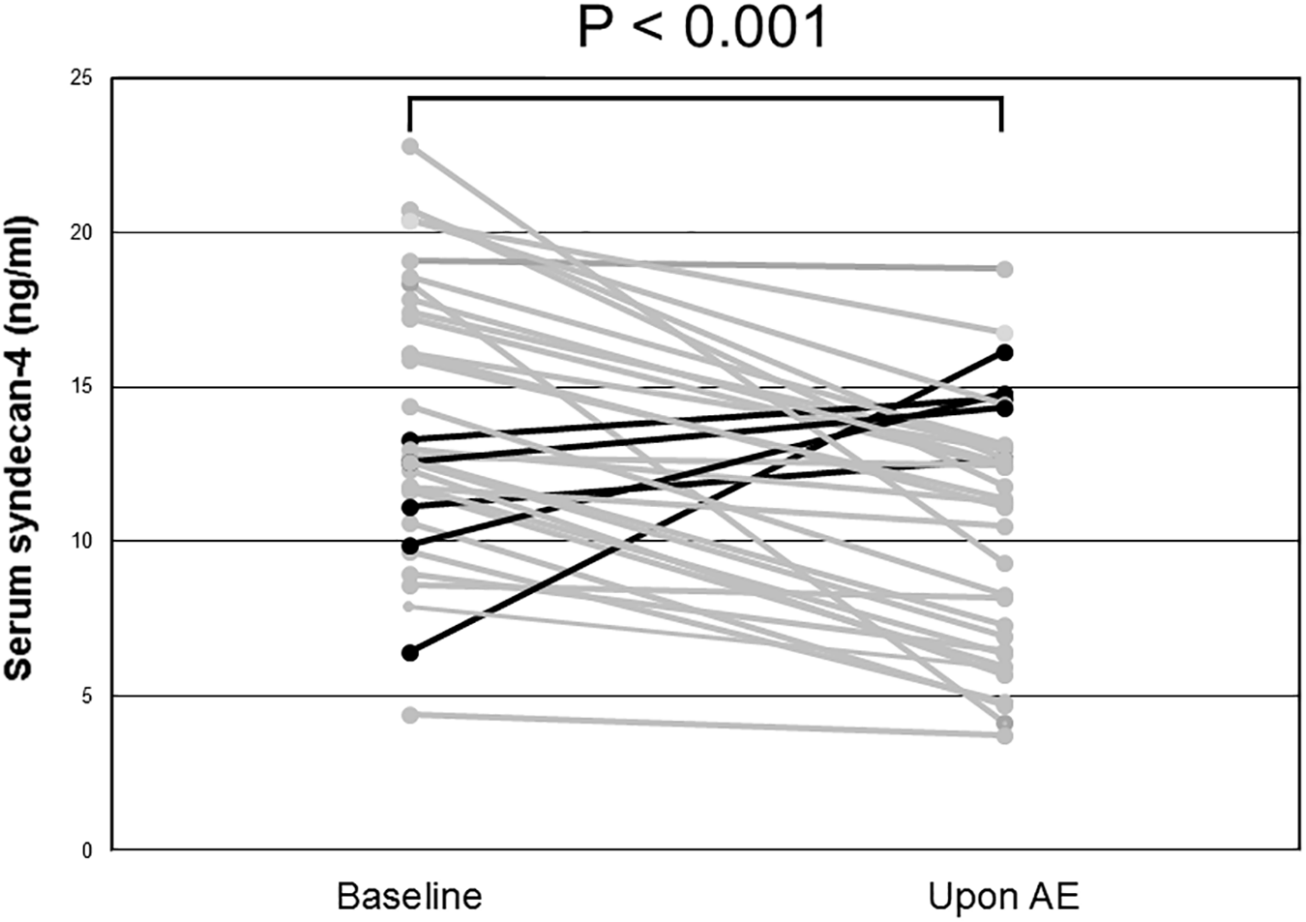
Changes in serum syndecan-4 levels of IIP patients before and upon AE. Serum syndecan-4 levels were compared in the same patients for whom analysis was possible during stability and upon acute exacerbation (n = 34). Serum syndecan-4 levels were significantly lower during AE than in the stable phase. AE: acute exacerbation. Wilcoxon’s signed-rank test was used for statistical analysis.

A comparison of serum syndecan-4 levels among the HV, SD-IIP, and AE-IIP groups revealed significantly higher serum syndecan-4 levels in the SD-IIP patients than in the HV and AE-IIP groups (25.22 ± 3.72 ng/mL vs. 16.05 ± 0.77 ng/mL vs. 10.65 ± 0.73 ng/mL; Fig 1). A comparison of the serum syndecan-4 levels between baseline and AE for 34 of the 56 AE-IIP patients for whom analysis was possible during stability revealed significantly lower serum syndecan-4 levels during AE than at baseline for the same patients (14.06 ± 0.78 ng/mL vs. 10.47 ± 0.69 ng/mL, p = 0.01 for baseline and AE, respectively; Fig 2). The mean time between blood sampling and AE was 556 ± 104 days.

### The relationship between serum syndecan-4 levels and clinical parameters

We also examined the correlation between the serum syndecan-4 levels of the AE-IIP patients upon admission for AE and their clinical parameters, including blood laboratory results, SIRS score, and APACHE II score. Serum syndecan-4 levels during AE were significantly positively correlated with WBC count and showed a weakly positive tendency to correlation with KL-6 and baseline %vital capacity (VC); however, no correlations were observed with SP-A or SP-D (Table 2). The possible correlation between serum syndecan-4 levels and clinical parameters was also analyzed separately in the IPF and non-IPF groups by first analyzing the correlation between serum syndecan-4 levels on admission and the clinical parameters evaluated in Table 2. The IPF patients (n = 21) showed results consistent with those of all AE-IIP patients, i.e., a significant positive correlation was observed between serum syndecan-4 levels on admission and WBC count. By contrast, analysis of non-IPF patients (n = 35) revealed a significant positive correlation between syndecan-4 levels on admission and KL-6 and baseline %VC and a significant negative correlation with PaO_2_. Division of the 34 AE-IIP patients with baseline syndecan-4 levels into a low baseline serum syndecan-4 group and a high baseline serum syndecan-4 group (based on a median baseline syndecan-4 level of 12.8 ng/mL) revealed no differences in the two groups for time from baseline to AE (423 ± 148 days vs. 688 ± 142 days) or frequency of use of pirfenidone (7/17 vs. 4/17), prednisolone (9/17 vs. 9/17), or immunosuppressants (2/17 vs. 6/17) during stability. The two groups also showed no differences in LDH (274 ± 11 IU/L vs. 282 ± 20 IU/L), KL-6 (1327 ± 171 U/mL vs. 1166 ± 167 U/mL), SP-A (106 ± 17 ng/mL vs. 84 ± 13 ng/mL), SP-D (269 ± 51 ng/mL vs. 217 ± 33 ng/mL), VC (2.02 ± 0.26 L vs. 2.04 ± 0.27 L), or %VC (64.3 ± 6.8% vs. 91.0 ± 30.1%; Table 3) during stability. No correlations were observed between the baseline serum syndecan-4 levels and the time to onset of AE. The baseline syndecan-4 levels also showed no correlation with LDH, KL-6, SP-A, SP-D, baseline VC, or %VC. The syndecan-4 levels also did not differ between IPF and non-IPF patients, in either the stability period (13.69 ± 4.63 ng/mL vs. 14.34 ± 4.62 ng/mL) or the exacerbation period (9.19 ± 3.86 ng/mL vs. 11.53 ± 6.17 ng/mL).

**Table 2:** Correlations of serum syndecan-4 levels on admission with clinical parameters.

	Correlation coefficients	P value
WBC	0.426	0.001
LDH	-0.074	0.587
KL-6	0.256	0.057
SP-A	0.079	0.569
SP-D	0.142	0.307
CRP	-0.157	0.249
ESR	-0.174	0.263
PCT	-0.104	0.573
BNP	-0.067	0.644
FDP	-0.161	0.285
D-dimer	-0.208	0.135
PaO_2_	-0.215	0.112
P/F	0.135	0.323
SIRS score	-0.209	0.123
Baseline VC	0.104	0.491
Baseline %VC	0.271	0.069
APACHE II score	-0.132	0.336

WBC: white blood cell count, LDH: lactate dehydrogenase, KL-6: Krebs von den Lungen-6, SP-A: surfactant protein-A, SP-D: surfactant protein-D, CRP: c-reactive protein, ESR: erythrocyte sedimentation rate, PCT: procalcitonin, BNP: brain natriuretic peptide, FBG: fibrinogen, FDP: fibrin and fibrinogen degradation product, PaO_2_: arterial partial pressure of carbon dioxide, P/F: arterial partial pressure of carbon dioxide/Fraction of inspiratory oxygen, SIRS: systemic inflammatory response syndrome, VC: vital capacity. APACHE II score: acute physiology and chronic health evaluation II score. Mean ± SEM.

**Table 3:** Clinical characteristics of idiopathic interstitial pneumonia patients with high and low baseline serum syndecan-4 levels.

	High baseline serum syndecan-4 group	Low baseline serum syndecan-4 group	P value
Subjects (n)	17	17	
Age (years)	71.3±1.6	67.9±1.9	0.120
Gender (M/F)	13/4	16/1	0.335
Duration until acute exacerbation from initial stable analysis (days)	423±148	688±142	0.352
Use of oral corticosteroid (+/-)	9/8	9/8	1.000
Use of pirfenidone (+/-)	7/10	4/13	0.465
Use of oral immunosuppressive agents (+/-)	2/15	6/11	0.225
LDH (IU/L)	274±11	282±20	0.787
KL-6 (U/ml)	1327±171	1166±167	0.517
SP-A (ng/ml)	106±17	84±13	0.327
SP-D (ng/ml)	269±51	217±33	0.885
Baseline VC (L)	2.02±0.26	2.04±0.27	0.664
Baseline %VC (%)	64.3±6.8	91.0±30.1	0.748

KL-6: Krebs von den Lungen-6, SP-A: surfactant protein-A, SP-D: surfactant protein-D, VC: vital capacity. Patients with patients with acute exacerbation of idiopathic interstitial pneumonia were stratified according to median serum syndecan-4 concentration (Low baseline serum syndecan-4 group; < 12.8 ng/ml. High baseline serum syndecan-4 group; ≥ 12.8 ng/ml). All blood laboratory parameters were analyzed at the time of blood sampling during stability. Mean ± SEM.

### The relationship between serum syndecan-4 levels and prognosis

We compared prognoses 60 days after admission for AE in the 56 patients with AE-IIP. After 60 days, the survival rate was 53.6% (30 patients). Comparison of the clinical characteristics of the survival (n = 30) and non-survival (n = 26) groups revealed higher FDP and APACHE II levels, lower baseline VC and %VC, and significantly shorter duration between initial stable analysis and AE for the non-survival than for the survival group (Table 4). The serum syndecan-4 levels upon admission for AE did not differ between the two groups, but the non-survival group had a significantly higher baseline serum syndecan-4 level (16.13 ± 1.12 ng/mL vs. 12.61 ± 0.97 ng/mL).

**Table 4:** Comparison of the clinical characteristics of survivors and non-survivors of AE-IIP.

	Survivors	Non-survivors	P value
Subjects (n)	30	26	
Age (years)	68.1±1.4	72.6±1.4	0.053
Gender (M/F)	28/2	19/7	0.066
IPF/non IPF (n)	14/16	7/19	0.170
Sivelestat (+/-)	9/21	14/12	0.103
Cyclophosphamide pulse therapy (+/-)	3/27	4/22	0.693
Duration of symptoms before admission (days)	9.8±1.3	7.8±1.7	0.046
WBC (/μl)	9880±756	11085±1006	0.215
LDH (IU/L)	378±31	532±115	0.092
KL-6 (U/ml)	1463±192	1594±165	0.286
SP-A (ng/ml)	109±9	132±11	0.174
SP-D (ng/ml)	277±29	427±111	0.663
CRP (mg/dl)	6.85±1.17	9.28±1.31	0.144
ESR (mm/h)	40±5	38±5	0.835
PCT (ng/ml)	0.21±0.78	1.23±0.59	0.012
BNP (pg/ml)	60.1±17.3	125.4±40.8	0.104
FBG (mg/dl)	433±22	476±22	0.178
FDP (μg/ml)	11.2±4.7	16.3±5.3	0.010
D-dimer (μg/ml)	5.9±2.1	7.4±2.4	0.084
P/F (mmHg)	238±23	168±20	0.051
SIRS (+/-)	17/13	17/9	0.589
SIRS score	1.8±0.2	2.0±0.2	0.677
APACHE II score	12.2±1.0	15.4±0.9	0.024
Baseline VC (L)	2.23±0.21	1.61±0.13	0.037
Baseline %VC (%)	84.3±17.6	53.3±3.9	0.032
Serum syndecan-4 upon AE (ng/ml)	10.54±1.11	10.78±0.95	0.657
Baseline Serum syndecan-4 (ng/ml)	12.61±0.97	16.13±1.12	0.042
Change in serum syndecan-4* (ng/ml)	2.30±1.01	5.43±1.11	0.142

AE: acute exacerbation, IPF: idiopathic pulmonary fibrosis, WBC: white blood cell count, LDH: lactate dehydrogenase, KL-6: Krebs von den Lungen-6, SP-A: surfactant protein-A, SP-D: surfactant protein-D, CRP: c-reactive protein, ESR: erythrocyte sedimentation rate, PCT: procalcitonin, BNP: brain natriuretic peptide, FBG: fibrinogen, FDP: fibrin and fibrinogen degradation product, P/F: arterial partial pressure of carbon dioxide/Fraction of inspiratory oxygen, SIRS: systemic inflammatory response syndrome, VC: vital capacity. APACHE II score: acute physiology and chronic health evaluation II score.

* Difference in serum syndecan-4 between before and upon AE in the identical patients. Survival was evaluated at 60 days after admission. All blood laboratory parameters were analyzed on admission. Mean ± SEM.

A univariate analysis of prognostic factors for survival 60 days after admission for AE revealed that age, P/F ratio, APACHE II scores, baseline VC, and baseline serum syndecan-4 levels were predictive factors for death (Table 5). A significant multi-collinearity was observed between P/F ratio and APACHE II scores, so we chose four variables (age, APACHE II scores, baseline VC, and baseline serum syndecan-4 levels) for multivariate analysis. This analysis revealed baseline serum syndecan-4 level alone as a significant prognostic factor (hazard ratio [HR]: 1.286, 95% confidence interval [CI]: 1.044–1.584; p < 0.05; Table 6).

**Table 5:** Univariate analysis of survival prediction. Univariate analysis.

Variable	HR	95%CI	P value
Age	1.085	1.005–1.171	0.037
Duration of symptoms before admission (days)	0.968	0.903–1.037	0.352
PCT	4.673	0.622–35.102	0.134
FDP	1.010	0.983–1.037	0.480
D-dimer	1.012	0.964–1.061	0.640
P/F	0.994	0.989–1.000	0.032
APACHE II score	1.142	1.017–1.283	0.025
Baseline VC	0.376	0.149–0.948	0.038
Baseline %VC	0.975	0.948–1.002	0.074
Baseline serum syndecan-4	1.216	1.015–1.458	0.034

PCT: procalcitonin, FDP: fibrin and fibrinogen degradation product, P/F: arterial partial pressure of carbon dioxide/Fraction of inspiratory oxygen, APACHE II score: acute physiology and chronic health evaluation II score, VC: vital capacity.

**Table 6:** Multivariate analysis of survival prediction. Multivariate analysis.

Variable	HR	95%CI	P value
Age	0.983	0.854–1.130	0.804
APACHE II score	1.123	0.911–1.385	0.278
Baseline VC	0.273	0.058–1.283	0.100
Baseline serum syndecan-4	1.286	1.044–1.584	0.018

APACHE II score: acute physiology and chronic health evaluation II score, VC: vital capacity.

**Fig 3:**
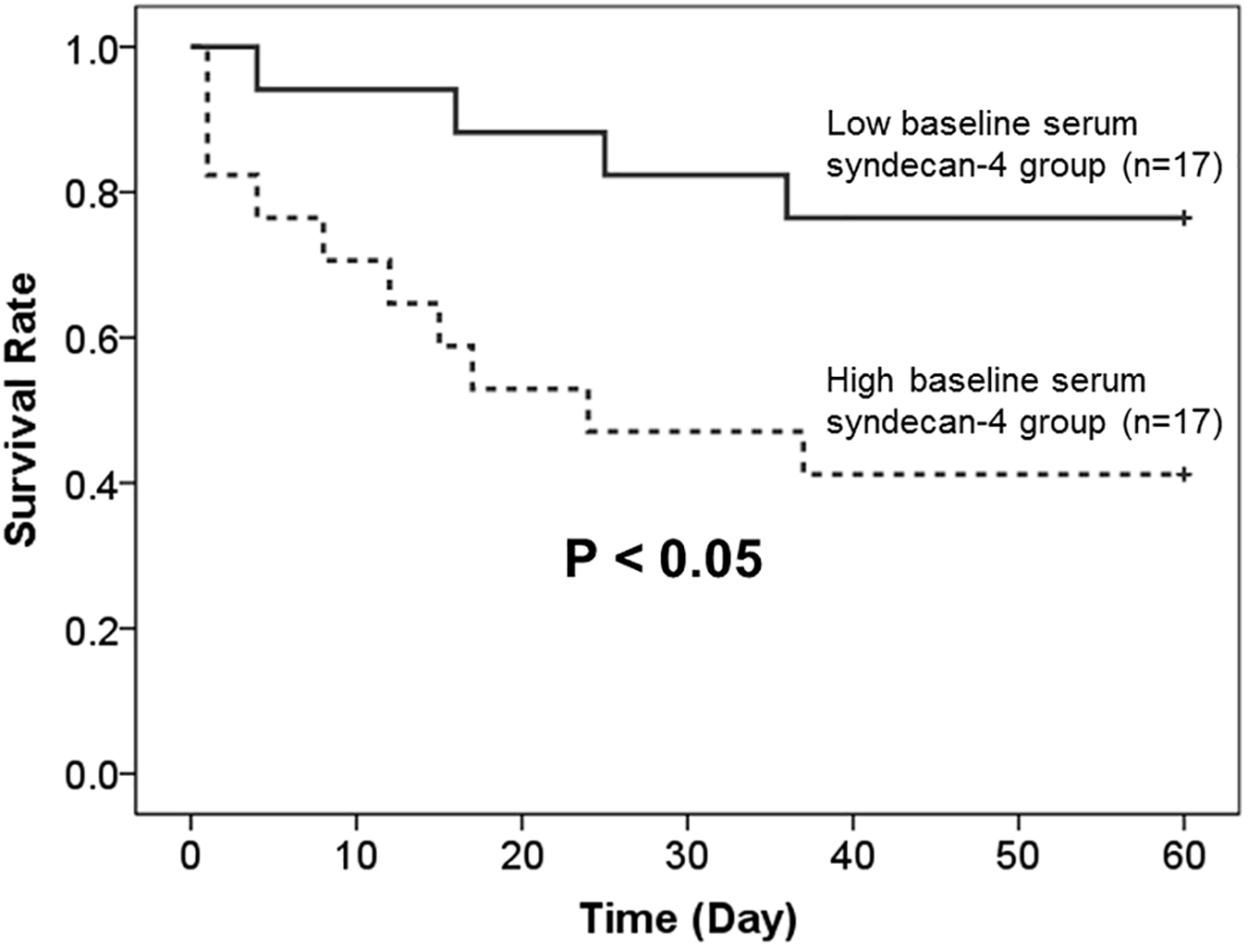
Kaplan Meier curves of the survival of patients with acute exacerbation of idiopathic interstitial pneumonia who were stratified according to median baseline serum syndecan-4 level. Baseline serum syndecan-4 levels measurements were obtainable for 34 of the 56 AE-IIP patients. The survival rate was significantly better for patients with low baseline serum syndecan-4 levels than with high baseline serum syndecan-4 levels. (Low baseline serum syndecan-4 group, < 12.8 ng/ml; High baseline serum syndecan-4 group, ≥ 12.8 ng/ml.) A log-rank test was used for statistical analysis.

Baseline serum syndecan-4 levels were available for 34 of the 56 AE-IIP patients; therefore, we divided these patients into high (n = 17) and low (n = 17) baseline serum syndecan-4 groups, using the median baseline serum syndecan-4 level (12.8 ng/mL) as a reference. As shown in Fig 3, prognosis was poorer for the high baseline serum syndecan-4 group. A further division of these 34 AE-IIP patients into groups with a high (n = 17) and low (n = 17) serum syndecan-4 upon AE, using the median serum syndecan-4 level (11.3 ng/mL) during AE as a reference, revealed no difference in prognosis. Analysis of all 56 AE-IIP patients also revealed no difference in prognosis between the groups with high (n = 28) and low (n = 28) serum syndecan-4 upon AE, using the median serum syndecan-4 level (10.6 ng/mL) during AE as a reference. Comparison of the prognosis between the IPF and non-IPF patients revealed no difference.

## Discussion

In the present study, we demonstrated that: (1) baseline serum syndecan-4 levels were higher in patients with IIP than in the HV group, and the levels decreased during AE; (2) serum syndecan-4 levels during AE were positively correlated with WBC count and showed weak positive tendencies with KL-6 and baseline %VC; (3) after AE, significantly higher baseline serum syndecan-4 levels were observed in the non-survival group than in the survival group; and (4) baseline serum syndecan-4 levels were the sole factor capable of predicting prognosis after AE in a multivariate analysis.

The AE of interstitial pneumonia is a clinically important event that influences the prognosis of patients with interstitial pneumonia. In IPF, AE occurs at frequencies of 8.6% and 23.9% at 1 and 3 years after diagnosis, respectively, with a reported post-onset mortality rate of approximately 50%. AE therefore accounts for 40% of all IPF-related deaths. AE also occurs in forms of interstitial pneumonia other than IPF [26, 27], with suspected involvement of viral infection, aspiration, exposure to air pollution, progression of abnormal fibroproliferation related to acute stress on the lungs, and other factors [28–30]. Increased neutrophil counts in bronchoalveolar lavage fluid [31], and histopathological findings of diffuse alveolar damage characterized by the formation of a hyaline membrane, similar to those of acute respiratory distress syndrome are observed [32–36]. However, organizing pneumonia and extensive fibroblastic foci can also be present, in addition to diffuse alveolar damage [37]. The presence of considerable epithelial injury has also been suggested due to higher levels of KL-6 and SP-D during AE of IPF than during stability [38, 39]. The levels of pro-inflammatory cytokines, such as CXCL1 and CXCL8/IL-8, may increase, together with the levels of M2 cytokines such as CCL18. M2 cytokines are considered to play an important role in wound healing, so these increases suggest strong upregulation of both lung damage and the wound healing process [40].

Much remains unclear about the pathogenesis of AE of IPF, although Kondoh et al. reported risk factors that included a decrease of at least 10% in a patient’s %VC value within 6 months, and an increased AaDO_2_ level at baseline [41]. Louis et al. reported a rise in plasma CXCL13 levels in IPF patients, higher CXCL13 levels in IPF patients with AE or in those who develop AE within 6 months, and poor prognosis when the CXCL13 level increases by 50% or more during the course of IPF, irrespective of the initial level, to cause subsequent AE-related respiratory failure [42]. Jenkins et al. also reported an association between increased degradation of extracellular matrix proteins by matrix metalloproteinases and disease progression, and that the rate of increase of degradation products was a predictor of survival [43]. However, no predictors of onset of AE of IPF or prognostic biomarkers after onset have been established thus far.

The members of the syndecan family of proteoglycans typically have three to five heparan sulfate and chondroitin sulfate chains, and exist as transmembrane proteins on epithelial cells, endothelial cells, macrophages, and fibroblasts in the lungs. Humans have four isoforms of syndecan (syndecan-1 to syndecan-4). Syndecan-4-deficient mice have a high mortality, as determined using mouse models of sepsis induced by intraperitoneal administration of LPS [44], and they show severe liver failure in response to concanavalin A [45], suggesting an important role of syndecan-4 in limiting inflammation. We also reported an anti-inflammatory effect of syndecan-4 in mice treated intratracheally with LPS [18] and with viable bacteria [19]. Arif et al. found that intravenous administration of recombinant syndecan-4 to mice increased bronchiolar progenitor cells and markedly decreased lung inflammation caused by naphthalene and bleomycin [46]. Jiang et al., using syndecan-4-deficient mice and a mouse model of bleomycin-induced pulmonary fibrosis, reported that syndecan-4 decreased fibrosis in the lungs by binding to CXCL10, a chemokine with an anti-fibrotic effect [20]. These findings suggest an important role for syndecan-4 in the pathogenesis of inflammation and fibrosis of the lung. In the present study, serum syndecan-4 levels during AE of IIP had a positive correlation with WBC count and weak positive tendencies with KL-6 and baseline %VC. The study population was divided into two groups, an IPF group and non-IPF group, and analysis of the correlations of serum syndecan-4 levels during AE and clinical parameters revealed differences between the two groups. In the IPF group, serum syndecan-4 level during AE was positively correlated with WBC, whereas in the non-IPF group, serum syndecan-4 level during AE had significant positive correlations with KL-6 and baseline %VC, and a significant negative correlation with PaO_2_. The precise cause(s) of these results is not evident, but the difference in the pathogenesis of acute exacerbation of IPF and non-IPF, if it exists, might explain the inconsistent results found between the IPF and non-IPF groups. The results presented here suggest that serum syndecan-4 levels reflect the degree of inflammation and fibrosis in AE of IIP. Although serum syndecan-4 levels decrease during AE, the precise cause is unknown because the source and kinetics of serum syndecan-4 in the human body have not yet been clarified. Excess secretion from the kidneys may be one cause of decrease in serum syndecan-4 during AE; Schmidt et al. have recently reported elevated levels of urinary glycosaminoglycans in patients with acute respiratory distress syndrome [47].

In the present study, the univariate analysis comparing factors between the survival and non-survival groups after the onset of AE revealed that the patients in the non-survival group were older and had significantly lower P/F ratio and baseline VC, and significantly higher APACHE II and baseline serum syndecan-4 levels. A multivariate analysis of survival based on the univariate analysis results also revealed that the baseline serum syndecan-4 level was the sole factor yielding a prognostic prediction after AE. The factors related to survival in AE of IPF reported thus far (SIRS score, LDH, KL-6, procalcitonin, severity of hypoxemia and degree of respiratory impairment prior to AE) [48–52] did not reveal associations with death; only baseline serum syndecan-4 level was a prognostic factor, which has interesting ramifications in terms of the pathogenesis of this disease. Patients with an elevated baseline serum syndecan-4 level likely had a poor prognosis during AE because, at the time of blood sampling during stability, syndecan-4 had increased in their lungs in response to a higher level of inflammatory and fibrotic activity of interstitial pneumonia. The resulting damage to the lung tissue would then prompt processes to relieve inflammation and promote repair in the lungs. However, our results showing an association between low levels of baseline syndecan-4 and better prognosis in AE-IIP patients seem to be inconsistent with this hypothesis. Syndecan-4 exists on the cell surface and is shed by proteases such as MMPs, resulting in two forms of syndecan; cell surface and soluble forms [14–16]. The exact kinetics of syndecan-4 protein in the human body have not been clarified, and we only analyzed serum syndecan-4 which is a soluble form. Inflammatory and fibrotic responses increase the expression of cell surface form of syndecan-4. We have previously reported that intratracheal instillation of LPS and viable bacteria into mice increases mRNA expression of syndecan-4 in the lungs [18, 19], and immunohistochemical analysis showed increased expression of proteoglycans in patients with pulmonary fibrosis including IPF [53]. Moreover, more shedding of membrane syndecan-4 must be induced in fibrotic lungs, because MMP levels are reported to be increased in pulmonary fibrosis [54, 55]. IPF patients with a high MMP-7 level are reported to have a poor prognosis [56], and our results are consistent with this. On the other hand, from the viewpoint of the role of syndecan-4 in IIP, elevated levels of syndecan-4, which possibly functions to inhibit excess pulmonary inflammatory and fibrotic processes, might not be sufficient to suppress excess inflammatory and fibrotic processes in IIP lungs. To clarify the exact role of syndecan-4 in the pathogenesis of pulmonary fibrosis, further investigations are necessary.

This study has some limitations. First, it was a retrospective study of IIP patients who had developed AE, so we did not analyze predictors of onset of AE following stability. Although we compared serum syndecan-4 levels in the same patients for whom analysis was possible during stability and upon acute exacerbation, the number of patients was limited. Our analysis of IIP patients did not show a relationship between the baseline syndecan-4 level and time to AE onset, so further study is required to determine if analysis of baseline serum syndecan-4 can predict the subsequent risk of onset of AE. If serum syndecan-4 proves to be a prognostic indicator of both the risk of AE onset as well as prognosis after onset, this would facilitate the development of treatment strategies, such as the consideration of aggressive therapy from an early stage in IIP patients who show an elevated baseline syndecan-4 level. This possibility should be evaluated by a prospective study. Second, we also need to consider the possible participation of bacterial pneumonia, as our previous study showed increased serum syndecan-4 levels under this condition [19]. We ensured an accurate diagnosis of AE by careful evaluation of various clinical findings, including sputum culture, urinary antigens such as *Streptococcus pneumoniae* and *Legionella pneumophilia*, serum β-D-glucan, and cytomegalovirus antigen. The results in our patients did not suggest the presence of respiratory infection and HRCT, conducted on all patients, did not show the typical radiological findings of bacterial pneumonia, such as segmental consolidations. However, exclusion of respiratory infection, or distinguishing respiratory infection from AE, can sometimes be difficult. In addition, some events, including respiratory infection, cause acute deterioration of interstitial pneumonia, and complete exclusion of triggered events is often difficult in the clinical setting.

A revised definition of AE was recently published by the international working group, which introduced a completely new term of “triggered AE” [8]. The report defined two types of AE: “idiopathic” and “triggered.” In the present study, procalcitonin levels, which have been reported to be increased in patients with AE-IIP [50, 57], were higher in the high baseline serum syndecan-4 group than in the low baseline serum syndecan-4 group. We cannot exclude the possibility that some of our AE-IIP patients may have had “triggered” AE. The potential differences in clinical characteristics, such as prognosis, have not been established for “triggered” and “idiopathic” AE, so further studies are required to identify these. Third, the mean age was also significantly lower in our HV group compared to the SD-IIP and AE-IIP groups. The relationship between age and serum syndecan-4 levels has not yet been investigated in healthy individuals, so final conclusions must await further studies.

Taken together, however, the results of the present study indicate that baseline serum syndecan-4 levels could be clinically useful as a prognostic indicator after the onset of AE.
